# Debate: Limitations on universality: the "right to health" and the necessity of legal nationality

**DOI:** 10.1186/1472-698X-10-11

**Published:** 2010-06-04

**Authors:** Lindsey N Kingston, Elizabeth F Cohen, Christopher P Morley

**Affiliations:** 1 Social Science Program, Maxwell School of Citizenship and Public Affairs, Syracuse University, Syracuse, NY 13244; 2 Department of Political Science, Maxwell School of Citizenship and Public Affairs, Syracuse University, Syracuse, NY 13244; 3 Department of Family Medicine & Department of Public Health and Preventive Medicine, S.U.N.Y. Upstate Medical University, 750 East Adams St, MIMC Suite 200, Syracuse, NY 13210

## Abstract

**Background:**

The "right to health," including access to basic healthcare, has been recognized as a universal human right through a number of international agreements. Attempts to protect this ideal, however, have relied on states as the guarantor of rights and have subsequently ignored stateless individuals, or those lacking legal nationality in any nation-state. While a legal nationality alone is not sufficient to guarantee that a right to healthcare is accessible, an absence of any legal nationality is almost certainly an obstacle in most cases. There are millions of so-called stateless individuals around the globe who are, in effect, denied medical citizenship in their countries of residence. A central motivating factor for this essay is the fact that statelessness as a concept is largely absent from the medical literature. The goal for this discussion, therefore, is primarily to illustrate the need for further monitoring of health access issues by the medical community, and for a great deal more research into the effects of statelessness upon access to healthcare. This is important both as a theoretical issue, in light of the recognition by many of healthcare as a universal right, as well as an empirical fact that requires further exploration and amelioration.

**Discussion:**

Most discussions of the human right to health assume that every human being has legal nationality, but in reality there are at least 11 to 12 million stateless individuals worldwide who are often unable to access basic healthcare. The examples of the Roma in Europe, the hill tribes of Thailand, and many Palestinians in Israel highlight the negative health impacts associated with statelessness.

**Summary:**

Stateless individuals often face an inability to access the most basic healthcare, much less the "highest attainable standard of health" outlined by international agreements. Rather than presuming nationality, statelessness must be recognized by the medical community. Additionally, it is imperative that stateless populations be recognized, the health of these populations be tracked, and more research conducted to further elaborate upon the connection between statelessness and access to healthcare services, and hence a universal right to health.

## Background

The "right to health" has been recognized as one of a set of basic human rights for at least the past half-century, since the adoption of the Universal Declaration of Human Rights in 1948. Currently, the "right to health" has been formally recognized by 56 national governments, in the form of constitutional or statutory provisions [1]. The scope and meaning of this right has been the subject of debate within the international community, however, and the means for achieving it remain similarly contested. The features of a healthcare system that is able to guarantee a comprehensive "right to the highest level of attainable health" for the citizenry of a given nation-state may be complicated by a variety of social and political obstacles. Backman and colleagues [1] recently reviewed the status of the right to health in 194 countries and found that much work still needs to be done before this right can legitimately be considered "universal."

Despite a general awareness within the scholarly community that the right to health may not be available universally, investigations of this right have in large part been limited in scope. This has two particular manifestations. First, debates frequently center on the structure, elements, and functions of health systems located within nations, thereby defining the state-centric platform from which citizens can access a right to health. An implicit assumption of this discourse is that the nation-state is the guarantor of first resort for the social rights of its recognized citizenry, either through state action or through the promotion of such efforts in the private sector. This assumption is codified for health rights by Article 12 of the International Covenant on Economic, Social and Cultural Rights (ICESR) [2]. Second, even when migration is addressed it is often from the perspective of those who migrate between nations by choice or as refugees. Both the citizens of a state who reside within its borders, as well as those who immigrate to a nation via politically legitimate channels of incorporation (e.g. legal immigrants and refugees), face a host of well-documented barriers to healthcare. Nevertheless, both recognized citizens and legal immigrants often do have access to these admittedly imperfect healthcare services because they receive political recognition from the public, private, or non-profit bureaucracies that govern and administer such services. In effect, both groups often have some access to a right to health within their nation of residence.

By contrast, individuals with no legal nationality - the right to reside somewhere and to be able to move freely - often have no venue in which to make claims to rights of health. In order to examine this issue we consider the case of statelessness, which has been defined as the condition by which someone "under national laws, does not enjoy citizenship - the legal bond between a state and an individual - with any country" [3]. The Office of the United Nations High Commissioner for Refugees (UNHCR) estimates there are roughly 11 million stateless people worldwide [3], despite international and domestic laws guaranteeing rights to legal nationality. Researchers at Refugees International describe their estimate of 12 million as conservative [4]. Although there are a number of factors that constrain a stateless person's ability to claim their right to health, lack of nationality often creates obstacles related to documentation, the inability to access national (and affordable) healthcare, and challenges related to mobility that decrease a person's chances of arriving at a hospital. Many stateless people have shorter-than-average life-spans as a result of vulnerabilities associated with lack of legal status; one stateless man recently described his plight as like being "buried alive" [5]. Lack of legal nationality, therefore, is directly related to a person's inability to secure effective *medical citizenship*, or the fulfillment of their right to health. Medical citizenship may be characterized as a subset of the larger category of social citizenship, through which basic standards of social welfare are enforced for a citizenry. Those without nationality cannot access institutional health provisions that are regularly secured by those recognized as belonging to a nation-state.

A central motivating factor for this essay is the fact that the concept of statelessness is largely absent from the medical literature. Even a systematic review specifically of the effects of statelessness upon health or access to healthcare is challenging; entering the term "stateless" into PubMed, for example, yields only 23 results in total, and only four from the past decade that are directly applicable to healthcare [6-9]. The goal for this discussion, therefore, is primarily to illustrate the need for further monitoring of health access issues by the medical community, and for a great deal more research into the effects of statelessness upon access to healthcare. This is important both as a theoretical issue, in light of the recognition by many of healthcare as a universal right, as well as an empirical fact that requires further academic exploration and public attention.

In order to illustrate this connection between nationality and medical citizenship we will first offer brief reviews of the right to health and the concepts of statelessness and nationality. Following this discussion, we then proceed to briefly illustrate selected instances of the effect of statelessness upon access to health. We will then conclude with recommendations for future action on this front.

### The Right to Health

The legal basis for the right to health is found within international law and agreements. The 1948 Universal Declaration of Human Rights (UDHR) contends in Article 25 that "everyone has the right to a standard of living adequate for the health and well-being of himself and his family," including medical care. Article 27 adds that everyone has the right to "share in scientific advancement and its benefits" [10]. More recently, the 2005 UNESCO Universal Declaration on Bioethics and Human Rights also states that "the enjoyment of the highest attainable standard of health" is a fundamental human right, and that "access to quality health care and essential medicines" is required "because health is essential to life itself and must be considered to be a social and human good." Furthermore, the UNESCO Declaration asserts that "the promotion of health and social development for their people is a central purpose of governments that all sectors of society share" [11].

Article 12 of the ICESR [2] entered into force in 1976, and recognizes the right to the "highest attainable standard" of health with specific provisions for the reduction of infant mortality and the prevention, treatment, and control of disease [2]. The most authoritative interpretation of Article 12 of the ICESR is General Comment 14 of the UN Committee on Economic, Social and Cultural Rights [12], which interprets Article 12 to be applicable to stateless populations in several direct ways. Paragraph 18 identifies and precludes discrimination based on a variety of factors, including "civil, political, social or other status," which has the intention or effect of nullifying or impairing the equal enjoyment or exercise of the right to health. Paragraph 34 describes legal obligations created by Article 12 to "*respect *the right to health by, *inter alia*, refraining from denying or limiting equal access for all persons, including prisoners or detainees, minorities, asylum seekers and illegal immigrants, to preventive, curative and palliative health services; abstaining from enforcing discriminatory practices as a State policy; and abstaining from imposing discriminatory practices relating to women's health status and needs," and Paragraph 43 describes the core obligations of states to ensure the right of access "to health facilities, goods and services on a non-discriminatory basis, especially for vulnerable or marginalized groups." Finally, Paragraphs 46 - 51 describe various acts of commission and omission that may be applicable to stateless populations.

General Comment 14 applies the tenets of Article 12 to stateless populations in several ways: it specifies duties and obligations of states to provide access to healthcare; it identifies the need for the distribution of the underlying determinants of health in an equitable and non-discriminatory fashion; it draws special attention toward vulnerable and marginalized populations; and it proscribes acts of commission (e.g., the stripping of citizenship or documentation from a person or group by law or fact) and/or omission (e.g., when populations are regularly excluded from birth registration) that may lead to statelessness. The right to health, therefore, has a strong legal basis in international law originating in these and other provisions. The question for stateless populations, however, is not whether they have the legal right to health or healthcare; but, rather, whether institutional safety nets enforce the legal articulation of these rights. Statelessness may therefore present a practical and fundamental paradox: in order to be able to exercise right to health accorded to them by international law, stateless persons must receive forms of political recognition from states that many states are often unable or unwilling to grant.

Despite solid legal underpinnings the medical citizenship actually available to individuals varies widely and depends upon a range of variables. Although some version of a "right to health" is perhaps the least normatively contestable social right, the specifics of what that right should or does entail are often difficult to determine. A right to health can include a number of components including: access to health services, resources necessary to achieve health, medical self-determination, the ability to resist conditions or policies that endanger health, health information and transparency, informed consent, and even a right to decision-making and accountability for health programs and policies [13]. The right to health, thus, must be specified in order to make it an enforceable right and not merely rhetoric. As one commentator points out, "a right to health that is too broadly defined lacks clear content and is less likely to have a meaningful effect," highlighting the importance of clear definitions that clarify state obligations, identify violations, and establish criteria and procedures for enforcement [14]. Furthermore, the right to health cannot be understood in a vacuum. Point 2 in General Comment No. 14 details that:

...the right to health is closely related to and dependent upon the realization of other human rights, as contained in the International Bill of Rights, including the rights to food, housing, work, education, human dignity, life, non-discrimination, equality, the prohibition against torture, privacy, access to information, and the freedoms of association, assembly and movement. These and other rights and freedoms address integral components of the right to health.

In short, the right to health cannot be realized in the absence of substantive and effective protections for a host of other human rights. As we discuss in the next section, of particular importance to stateless persons is non-discrimination and movement.

In response to concerns about indeterminacy and accountability, transnational actors have introduced new standards for advancing health as a human right and for specifying what this right confers. Advocates of health rights - which include professional associations, intergovernmental organizations, networks of patients and human rights organizations, and prominent individual activists such as Paul Farmer - combine protections of the person (such as the right to refuse medical treatment or an occupying power's responsibility to allow healthcare for citizens and prisoners of war) with social rights (such as the right to be provided the highest level of care attainable within one's society) [15]. The World Health Organization (WHO), for example, prepares a biennial list of essential medicines that outlines the core needs for a basic healthcare system and advocates for universal access to such medications [16]. Physicians for Human Rights recommends the highest attainable standard of health through access to skilled health workers and essential medications, as well as draws attention to underlying determinants of health such as clean drinking water and health education [17]. The need to address the underlying determinants is also clearly articulated in Article 12, and further annunciated and defined in General Comment 14, as described above.

This framing of health as a universal right, like all human rights, imposes three obligations on states. First, states must respect this right; states may not interfere with the enjoyment of the right to health, either directly or indirectly. Second, states must protect this right by taking measures to prevent private persons and businesses from interfering with the right to health. Third, states must fulfill the right by facilitating and promoting medical citizenship [14]. However, Farmer notes that those most likely to be denied access to healthcare are vulnerable populations most at risk for other human rights abuses and structural violence such as poverty and social inequalities based on race or gender [18]. As the following discussion will highlight, many of these individuals are vulnerable due to a lack of legal status that serves as a requirement for people to access their right to health.

### Legal Nationality

In the aftermath of two world wars and the consequent dissolution and realignment of states, the Universal Declaration of Human Rights was established in 1948 [10]. Article 15 of the Declaration included a right to a nationality and prohibition of arbitrary deprivation of citizenship. The development of a legal framework for the protection of nationality rights occurred gradually over the two decades that followed the Declaration. An excellent account of the development of nationality rights during the post - World War II decades appears in Dorothy Jean Walker's scholarship [19]. During the 1950 s, draft conventions on the Elimination of Future Statelessness and on the Reduction of Future Statelessness slowly elaborated upon the rights of the stateless. Most notably:

• The 1951 Convention Relating to the Status of Refugees extended protections to stateless refugees, including the right to asylum;

• The 1954 Convention Relating to the Status of Stateless Persons extended some protections to stateless individuals who were not refugees; and

• The 1957 Convention on the Nationality of Married Women, Article 1, protected a woman's citizenship in the event of divorce or marriage to a foreign national.

The most influential piece of international law related to statelessness, however, was the 1961 Convention on the Reduction of Statelessness. In Articles 1-4, the Convention attacked the problem of statelessness at birth by requiring states to grant nationality to all children born in its territory who would otherwise be stateless. Article 5(1) attempted to eliminate change of status - such as marriage, divorce, and adoption - as a cause of statelessness, and Article 8(1) sought to contain discriminatory denationalizations that gave rise to statelessness. Article 10 afforded some protection from statelessness arising out of transfers of territory. However, the Convention took a full thirteen years to garner the necessary number of ratifying or acceding states in order to enter into force, with Walker noting that the slow pace of ratification probably reflected "the reluctance of states to give up a measure of sovereign autonomy over nationality within areas of domestic jurisdiction" [19].

An important reason for addressing statelessness stems from the connection between rights protections and nationality. Writing after World War II, Hannah Arendt criticized conceptions of human rights for their inability to protect those who lacked citizenship and the protection provided by a nation-state, noting that "it turned out that the moment human beings lacked their own government and had to fall back upon their minimum rights, no authority was left to protect them and no institution was willing to guarantee them" [20]. More recently, scholars of citizenship have criticized both the UN and the UDHR for placating state needs at the expense of individual rights. For example, Seyla Benhabib has described not only a tension but an "outright contradiction" between rights and states' sovereign claims, citing that "a series of internal contradictions between universal human rights and territorial sovereignty are built into the logic of the most comprehensive international law documents in the world" [21]. Thus, even though the framing of today's social contract may have changed to reflect universal rights-based language, the power to grant and protect those rights remains in the hands of the nation-state.

The community of European states has attempted to eliminate these rights inequalities through the creation of the European Court of Human Rights, as established by the European Convention on Human Rights (ECHR) [22]. The ECHR, which entered into force in September 1953, secures fundamental civil and political rights to everyone living within state party jurisdictions. By the court's own public claim, all people, not just nationals, are included in the Convention [23]. Despite its idealistic promise, however, the ECHR is plagued by some of the same problems as the UDHR. In addition to being overwhelmed by applications that threaten to "paralyze the Convention's judicial process," seemingly "universal" rights are better defended by those with wealth and social standing - goods that stateless populations generally lack [24]. Although members of vulnerable populations have a chance of being heard in court, ultimately "a privileged applicant has far greater chances to be heard by the Court than an underprivileged one" [25]. Despite the promise of universality exhibited by the ECHR and the European Court, therefore, rights protections for non-nationals are not guaranteed. In fact, increasing violence and racism against the Roma - many of whom make up Europe's largest stateless population - has been observed throughout the European Union, including cases of expulsion, killing, and arson [26].

This disconnect between human rights law and protection not only leaves millions of people stateless, but also threatens the right to health and a variety of other rights as outlined in international law. Ultimately, the relationship between health rights and legal nationality is framed by the larger concept of citizenship. Citizenship is an omnibus term comprising legal rights, status, and activities associated with formal political membership. T.H. Marshall's work provides the starting point for most contemporary discussions of citizenship [27]. In his seminal essay, Marshall describes a historical evolution of citizenship since the 18^th ^century, the trajectory of which culminates in the braiding together of civil, political, and social rights. Over the past half-century, this process unfolded in international law courts and venues. Civil rights preserve individual liberties such as contract, property, speech, conscience, while political rights ensure representation and participation. Social rights exist to prevent the worst effects of capitalism from rendering civil and political rights meaningless. Social rights such as pensions and welfare protect people's rights to a decent standard of living, for example, while education allows people to gain the enlightened understanding required for self-representation [28]. The social rights encompassed by healthcare provisions protect medical citizenship, fulfilling the right to the standard of health required for social participation. An additional concept that is useful to consider in the context of statelessness is that nation-states have often recognized differences in the nature or situation of particular groups within their populations. In doing so, they create "semi-citizens," who enjoy more limited sets of rights and/or protections than citizens [29]. Semi-citizens encompasses the diverse group of persons, including but not limited to migrants, who are eligible to enjoy some but not all of the rights of full citizenship described above. Whereas formal recognition as a legal migrant, refugee, or other form of semi-citizen brings with it *de facto *as well as *de jure *rights in piecemeal fashion, it is often empirically the case that those without legal nationality (or the ability to demonstrate legal nationality through formal documentation) do not have, or cannot access, even limited packets of rights on the local or national level, despite international recognition and pronouncement of basic human rights and right to a nationality.

Marshall was relatively inattentive to matters of place and the questions raised by displacement and immigration. Thus rights associated with legal nationality did not figure in his triad. Yet in today's increasingly mobile world these rights are not only as important as civil, political, and social rights but, in some circumstances, they will also govern a person's ability to access the rights Marshall considered fundamental to citizenship. Despite the development of limited free-movement zones, in general nation-states still monopolize the conferral of documents such as passports and greencards/work permits that in turn allow people to live within a set of borders, as well as documents such as visas that permit people to cross borders. Formal, state-issued documents are often utilized not just for internal or external mobility but also for general identification. This was apparent in the 2003 decision by the ECHR in Smirnova v. Russia, in which a Russian citizen had her passport revoked, and could not receive health services without this document [23]. Although Smirnova was not truly stateless, the end result was the same as if she were. By definition, a stateless person has no state in which to lodge a claim for rights, including the right to health. This lack of legal nationality deprives individuals of their medical citizenship, along a myriad other rights.

The consequences of the connection between legal nationality and medical citizenship are wide-ranging and deeply consequential. Non-nationals are often denied access to healthcare, or provided lower standards of care as compared to their citizen counterparts, and research suggests patterns of barriers and limited access to care for undocumented noncitizens. In California, for example, undocumented Latino immigrants have significantly worse healthcare experiences than their documented and US-born counterparts - including fewer sources of usual care, more difficulty understanding medical information, and a belief that they would receive better care if they were a different race or ethnicity [30]. In addition, not being a U.S. citizen was found to be a barrier to receiving cervical and breast cancer screening and citizenship status is correlated with access to health insurance, receipt of medication, and referrals to mental health services [31]. Although these immigrants usually hold legal nationality in a home country and are therefore not stateless, their experiences highlight the correlation between healthcare and citizenship. In countries with national health plans and subsidized services, lack of nationality often places medical citizenship outside the reach of stateless individuals, many of whom do not have documentation or adequate economic resources. Farmer notes that socialized healthcare often represents "strategies of the affluent" because it bars (poor) noncitizens from access to health services while (wealthy) citizens rarely criticize such practices [18]. Many countries, including European powers such as France and the Scandinavian countries, spend a great deal of resources preventing noncitizens from accessing healthcare in these situations of national systems [18]. These obstacles to healthcare lead Farmer to criticize modern health systems, arguing that "access to the fruits of science and medicine should not be determined by passports, but rather by need" [18]. Furthermore, health rights cannot be provided only to people of certain nations; people should not be erased by the "geographical chance" of living beyond the borders of an affluent state and consequently barred from receiving healthcare [18].

Although citizenship alone cannot guarantee equal standing within a society, legal status is nonetheless a prerequisite for the *possibility *of attaining such equality. This concept has been central to theorists of citizenship for at least the last half-century. Theories of rights and citizenship formulated by scholars such as Marshall [27] and Rawls [32-34] are both situated firmly in the context of the nation-state. The need for citizenship to access rights is not only conceptual or theoretical, but empirically observable as well, as field reports generated by advocacy organizations such as Refugees International [35,36] repeatedly find that stateless populations often exist in an "international blind spot," which refers to the difficulty of tracking and enforcing the rights of those who exist without official ties to any nation-state [37]. Herself a formerly stateless refugee, Arendt acknowledged this simple but crucial fact, criticizing the "abstract nakedness of being nothing but human" that leaves one vulnerable to rights abuses [20]. Walker further elaborates that legal nationality would be of less fundamental importance "if the status of statelessness were in fact given international recognition and protection" and political rights were extended to all people living within state boundaries. However, she warns that "this is not the reality of our nation-state organized world where [citizenship] is the linkage of the individual to right" [19].

Although there is a growing body of international law, as described in the preceding sections, that articulates fundamental rights that ought to be enjoyed by stateless populations, the language of these documents (such as General Comment 14) acknowledges that the nation-state remains the most effective enforcer of even international human rights, a fact also noted in the academic literature by postnationalists such as Soysal [38] as well as their critics [39]. If we are to view human rights such as health as the fundamental basis for living a good life, we must similarly view legal status as a fundamental necessity for claiming those rights. For, as in the words of Arendt, "no paradox of contemporary politics is filled with a more poignant irony than the discrepancy between the efforts of well-meaning idealists who stubbornly insist on regarding as 'inalienable' those human rights...and the situation of the rightless themselves" [20].

## Discussion

Legal nationality fulfills the "right to have rights" [20]. That is, one is legitimized by their membership in a state-centered political community and loses crucial political recognition when nationality is withdrawn or withheld. Without such membership, individuals will almost certainly face grave challenges in accessing their right to health, including the ability to secure basic healthcare and essential medicines. Because our modern political system organizes human beings into nation-states with demarcated boundaries, citizenship of a state is often a prerequisite for claiming ostensibly universal human rights. Following massive displacements and growing statelessness after the World Wars, Arendt noted that such "supposedly inalienable" rights proved unenforceable "whenever people appeared who were no longer citizens of any sovereign state" [20]. For this reason, statelessness has devastating consequences for the health of persons included within this category.

Academic discussions of the human right to health often assume that every human being has legal nationality. Largely absent from such discussions is a consideration of individuals who have no nationality claims anywhere: stateless individuals who lack any legal relationship to a nation-state. These individuals are deprived of nationality as a result of a "bewildering series of sovereign, political, legal, technical or administrative directives or oversights," including: arbitrary deprivation of nationality by the state, conflicts of law, procedural problems such as excessive fees or unrealistic deadlines, and failure to register a child at birth [3]. Whereas academic discussions of the human right to health assume that every human being has legal nationality, international human rights law explicitly rejects such assumptions. Article 2 of the UDHR states that

Everyone is entitled to all the rights and freedoms set forth in this Declaration, without distinction of any kind, such as.... national or social origin... birth or other status.

In Article 2.2 of the International Covenant on Economic, Social and Cultural Rights, states commit to guarantee the Covenant's rights "without discrimination of any kind," including discrimination based upon national or social origin, or birth status.

However, the UNHCR's own studies of the efficacy of its human rights doctrines, and in particular the UDHR, have repeatedly demonstrated the difficulties associated with the special case of the stateless. For example, UNHCR's "Report on the Implementation of the 1954 Convention within the European Union Member States" formulated recommendations for harmonization, indicating that:

...most States have not put in place specific mechanisms which will allow the identification and recognition of stateless persons...The possibility for a stateless person to secure residence will often be the necessary prerequisite for him or her to exercise the rights provided for by the Convention, which for the most part are only applicable to persons lawfully staying in the country. States are therefore invited to introduce mechanisms to promote the acquisition of lawful stay in appropriate cases for recognized stateless persons, in particular for those who have no other option.

Signatory states remain unable to identify stateless populations, as most states do not have mechanisms that effectively identify cases of statelessness within their own borders [40]. This, combined with an apparent reluctance to offer stateless persons access to a full panoply of human rights, has led to the conclusion that human rights law has not yet successfully transcended the barrier of statelessness. While there are ample and prominent acknowledgements of the rights of stateless persons in key statements on international human rights, these assertions have yet to achieve a level of applied efficacy that meets the standards of the organizations that have issued them. A recent evaluation noted that despite the steady increase of accessions in the last ten years, the statelessness conventions have still not been ratified by a sufficient number of states for them to have a truly significant effect on reducing statelessness and protecting stateless persons [40]. Furthermore, the main thrust of UN efforts with respect to statelessness, and evaluations of the efficacy of documents asserting the rights of the stateless, has been directed at reducing and eliminating statelessness rather than asserting the rights of the stateless [41]. In important respects this is a tacit acknowledgement of the dire challenges that come along with trying to accord stateless persons equal rights with citizens and, more generally, with justly governing the stateless. More specifically, it calls attention to the as yet unrealized goals expressed in which non-discrimination and free movement, among other rights, are identified as necessary to the realization of health rights for stateless persons [12]. Furthermore, this approach skirts thorny ethical questions associated with populations that explicitly seek to avoid the imposition of legal nationality. Because of the obstacles outlined in the preceding section, those without nationality are often unable to access specific services and medicines usually available to full citizens. The impacts of these obstacles are reflected in overall poorer health, including higher rates of infection and chronic illness, as well as increased mortality. As noted above, statelessness has been directly tied to obstacles related to documentation, the inability to access healthcare, challenges related to mobility, and shorter-than-average life-spans resulting from the inadequacies associated with lack of legal status. The condition of statelessness is often described as legal invisibility, due in part to the inherent lack of state protections and services that include access to healthcare.

In addition to the status of nationality, individuals also generally require documentation of nationality in order to claim an array of human rights, including the right to health. For stateless persons, acquiring such papers is often impossible. Politics is increasingly governed by "documentary nations," or document-requiring societies which require papers such as passports and identity cards to access social goods ranging from education and healthcare to entertainment and mobility [42]. Sometimes such documentation is explicitly denied to excluded groups, as was the case in Thailand when the Ministry of Interior directed district officials not to register the births of hill tribe children in 2002 [43]. In other cases, weak governments simply lack the resources to effectively document their populations. Without proof of nationality many individuals cannot access even the most basic of health services. Lack of documentation (often resulting from ineffective systems of birth registration) plays a significant role in childhood deaths from preventable diseases, which impact millions of children each year from birth to age five. Stateless children may be denied services, including subsidized vaccination programs, or may be required to pay more than patients with citizenship, a circumstance that often erects insurmountable financial barriers [44]. Children without birth certificates cannot be legally vaccinated in at least 20 countries, and more than 30 states require documentation to treat a child at a health facility [35]. The availability of documentation has also been cited as a factor reducing the risk of childhood exposure to HIV/AIDS, since identification papers firmly establish a child's age and make them less susceptible to early marriage agreements and sexual exploitation; in Uganda and Zambia, for example, birth certificates are considered key for establishing police protection of children at risk for human rights violations. According to Unity Dow, a High Court judge in Botswana, "[A] person who lacks proof of identity is, in the eyes of officials, a non-person" [45]. In other words, statelessness is a condition that can arise not only in circumstances of conflict and displacement, but also where state bureaucracies are not able to maintain a monopoly on the administrative facets of citizenship. For the purposes of individuals who need to claim rights such as the right to health, weak states produce an effect similar to ascriptive exclusion or war-induced displacement. At the end of the day each leaves people without legal nationality.

Despite these connections between nationality and the right to health, the medical community has largely overlooked the problem of statelessness. There are numerous clusters of stateless populations around the globe, including clusters in Côte d'Ivoire; Sahrawis taking refuge in Algeria; the Banyaumulenge in the Democratic Republic of Congo; Eritreans in Ethiopia, Nubians in Kenya; the Rohingya in Burma; ethnic "hill tribes" in Thailand; Bhutanese refugees in Nepal; Palestinians, Kurds and the Bidun (also Bidoon or Bidoun) throughout the Middle East; the Roma in some parts of Europe; Meshketians in Russia; a variety of other groups throughout former Soviet republics; and numerous ethnic groups across Africa and, to a lesser extent, Latin America [36]. In many cases, these groups are absent by name from the medical literature or are mis-described with the use of terms like refugee. Arguments for a connection between the nationality of such groups and their access to health services are absent. Most discussion centers around the provision of specific or general medical services in *ad hoc *fashion to ameliorate the immediate or long-standing consequences of displacement. However, there is little or no discussion of remediation via the recognition of a standing right to health, and the consequent responsibility of the host nation to provide such services - in effect, to recognize the medical citizenship claims of such groups. In the next section we present three brief case studies that illustrate the potentially direct connection between legal nationality status and access to healthcare: the Roma of Europe, the hill tribes of Thailand, and Palestinians in Israel.

### European Roma

The Roma, a linguistically and religiously diverse population that migrated to Europe in several waves from northern India over the past millennium, represent a major stateless population throughout Europe, particularly in the former Soviet bloc states. Although information on Roma populations is often limited and varies by country, many (but not all) Roma lack legal nationality [36]. In many cases, desperately poor and ethnically marginalized Roma populations have been excluded from full citizenship by a patchwork of laws, as well as by circumstances that prevent full documentation [46]. For some Roma, it may also be that documented legal nationality imposes costs and requirements that they regard as burdensome and intrusive on their own political identity [47]. These situations occur despite the adoption of the 1997 European Convention on Nationality and the 2006 Convention on the Avoidance of Statelessness in relation to State Succession, as well as the aforementioned international agreements. Based on United Nations estimates, the Council of Europe estimates that there are currently 679,000 stateless individuals in Europe; the Roma makes up a significant portion of this population [36].

The consequences of Roma marginalization (including poor educational opportunities, poverty, and stigmatization) certainly contribute to severely compromised health outcomes in essentially all Roma populations [46,48-50], but it is important to also note the core problems of documentation and citizenship status impacting this group's ability to access rights in general. The European Roma Rights Center has recently reported a number of barriers to accessing documentation, including population displacement after the Balkan wars in the 1990 s, relatively low birth registration rates, difficulty in replacing lost citizenship documentation due to cost or illiteracy, and simple obstruction and arbitrary decision-making on the part of granting officials. Beyond these highly specific causes, more general citizenship policies have tended to draw citizenship lines around the majority population in many recently unfederated nations, to the exclusion of minority groups such as the Roma [46].

The combined effects of these issues, which essentially deny nationality to many Roma, have downstream effects upon the right to health. For example, Boika and colleagues conducted a qualitative study of Roma healthcare seekers and policymakers in Bulgaria, and found that changes in one's place of residency and/or a lack of identity documents resulted in the inability to register with a physician in order to obtain health services [51]. The European Committee of Social Rights ruled in April 2009 that Bulgaria was in violation of the European Social Charter by failing to meet its obligations related to providing Roma populations with adequate access to healthcare. The Committee found that "significant cases of discriminatory practices against Roma in provision of medical services" was occurring throughout Bulgaria, including government restrictions on health insurance and social assistance as well as a lack of systematic measures to promote health awareness [52].

Similar problems facing Roma populations are prevalent throughout Europe. In Macedonia, for example, many Roma are explicitly excluded from Macedonian citizenship, and hence from state health insurance [46]. In Romania, inadequacies related to Roma health have been linked to a lack of health insurance, specialized medical personnel, adequate medical infrastructure, doctors' goodwill, and basic information on fundamental rights. The exclusion of Roma from the national healthcare system is reflected in statistics illustrating high rates of premature births and infant mortality, chronic measles and tuberculosis foci, lice infestations, and a life-expectancy well below the national average [53].

### The Hill Tribes of Thailand

The stateless population of Thailand is currently estimated to number 2 to 3.5 million, despite efforts on the part of the Thai government to grant nationality to some members of "hill tribe" ethnic minorities. Part of the cause of Thai statelessness lies in other policies that contradict the nationality-granting efforts. As noted previously, policy decisions to not register the births of hill tribe children thwarts recognition of the existence of these individuals, and hence makes granting of nationality all the more difficult [43]. Additionally, many members of the Karen and other hill tribes have been displaced over several generations of war in neighboring Burma, and their nationality is not recognized by either state [43,54].

Lack of nationality and the resulting absence of documentation prevent stateless persons from accessing affordable healthcare in Thailand. The government introduced a subsidized "30-baht plan" in 2001 with the intention of universalizing access to healthcare. In order to take part in the program, individuals must present identity documents to local administering officials in order to receive a "gold card" that ostensibly allows them to obtain basic health services for a fixed fee of 30 baht, or roughly US$0.88. The program has covered nearly 14 million people who were previously uninsured [55], yet roughly 4.4% of the population still lacks health insurance. Since its inception in 2001, a primary reason that people are denied access to the 30-baht plan is a lack of identifying documents [55,56], which is a particular problem for the stateless population of Thailand. Without documentary proof of Thai citizenship, an individual cannot access affordable healthcare under the 30-baht national program.

While there is no established causal relationship between statelessness and poor health in the Thai population, it is probable that lack of legal nationality stands in the way of access to health services and coverage [54]. As nationality begets documentation, and documentation begets access to the 30-baht plan, the lack of nationality amounts to a denial of basic health services that are available to all formally recognized Thai nationals. The effects of this denial are magnified for otherwise vulnerable populations, such as children and women of childbearing age. For example, the rate of child malnutrition is much higher among hill tribe children than it is for their urban, more fully enfranchised peers. These children also tend to have comparatively high rates of conditions associated with nutritional deficiencies, such as scabies, diarrhea, and lung infections [57,58]. Physicians for Human Rights additionally links lack of nationality to the denial of reproductive health services for women and girls [43].

Conflict within neighboring Burma has also contributed to the troubled health situation of hill tribe members in northern Thailand. Members of the Shan minority, for example, have been driven across state lines as a result of widespread abuses by the Burmese military regime. Denied refugee status or nationality in Thailand and often recognized only as enemy "insurgents" in Burma, these stateless individuals are often forced into exploitative situations (such as the Thai sex industry) and are simultaneously denied basic healthcare services. Data on stateless Shan migrants in Thailand indicates that this group bears a disproportionately high burden of infectious diseases, especially HIV, tuberculosis, lymphatic filariasis, and some vaccine-preventable illnesses. Not only does this situation undermine the right to health, it also undermines Thailand's ability to control many infectious diseases that may spread throughout broader populations [58].

### Palestinians

The complicated case of Palestine is a further illustration of the relationship between legal nationality and the right to health. Palestinians, who represent the world's largest stateless population with more than four million stateless people located throughout several countries [59], often suffer negative health consequences due to their inability to freely travel to hospitals and access medical supplies. Israeli immigration and citizenship policies have been cited as human rights violations due to inherent discrimination based on race, and stateless Palestinians residing within the Israeli state's borders face detention as illegal residents [60]. It is important to note that not all Palestinians are stateless, however. The term "stateless" here implies that some Palestinians have not secured legal nationality of an established state (such as Israel, Lebanon, or Egypt), not that this group currently lacks Palestinian statehood.

Stateless individuals in Israel have severely compromised and irregular access to national health insurance and social services. Although Palestinians residing within the 1967 state borders usually retain Israeli nationality and are able to access rights, permanent residents residing in the outskirts of Jerusalem and Palestinians living in the occupied territory do not have such state protections [59,61]. They are at risk for interruptions in access due to policy shifts, such as when the Israeli Ministry of the Interior revoked residency status of Palestinians residing outside of Jerusalem, confiscated ID cards, and deprived these individuals of health services, national insurance allowances, and rights of movement [61]. Some may access humanitarian aid via the United Nations Relief and Works Agency for Palestinian Refugees in the Near East (UNRWA), which means that many stateless Palestinians are not covered by the 1954 Convention Relating to the Status of Refugees (since those receiving UN assistance are not covered by the statelessness convention); those who have not attained Israeli or other nationality, however, remain stateless and often suffer the consequences of their lack of effective legal status [36].

Although standards of health are generally higher in the occupied Palestinian territory than in several other Arab countries, they are substantially lower than in Israel [62]. Health services for Palestinians in the occupied territory were neglected and under-funded by the Israeli military administration between 1967 and 1993, resulting in shortages of staff, hospital beds, medicines, and a range of services. Independent Palestinian health services have since been developed in an attempt to fill the gaps, yet they often lack health personnel (especially in areas such as family medicine, surgery, psychiatry, and nursing) and fail to meet consistent standards for training, equipment, and overall quality [62]. These shortcomings have direct consequences on health indicators; for instance, infant mortality rates stalled at around 27 per 1000 from 2000-06, the same as in the 1990 s, and indicate a slowdown of health improvements, a possible increase in health disparities, or an indication of deteriorating conditions. Stunting in children, an indicator of chronic malnutrition and increased disease burden, has risen from 7.2 percent in 1996 to 10.2 percent in 2006. Additionally, incidences of pulmonary tuberculosis, meningococcal meningitis, and mental health disorders have also risen in recent years [62].

Many Palestinians rely on only six hospitals for routine, emergency, and specialized treatments. Yet, the difficulties in securing necessary travel permits to drive to those hospitals have led to reductions in patient admissions by 50 percent [61]. Researchers contend that denied or delayed passage at government checkpoints have significantly affected access to civilian medical care, and that Israel's prior closure of access to the Gaza Strip seriously impeded operation of clinics and hospitals there. At least 68 pregnant Palestinian women have given birth at Israeli checkpoints since the beginning of the second Intifada in September 2000, resulting in at least 34 miscarriages and the deaths of four women [63]. A wall built to divide the West Bank from the rest of Israel has also impeded access to medical care, particularly for Palestinians living in the closed zones between the Wall and the Green Line; in that most vulnerable region, 79 percent of families are separated from health centers and hospitals [63]. Unable to attain effective nationality and separated from health services allocated to the Israeli polity, many Palestinians cannot access their right to health.

## Summary

As this brief discussion has illustrated, legal nationality serves as an often unstated prerequisite for accessing medical citizenship and the human right to health. Those lacking legal status - including Roma populations throughout Europe, hill tribe members in Thailand, and stateless Palestinians in Israel - frequently face an inability to access the most basic healthcare, much less the "highest attainable standard of health" outlined by the ICESR. Although the right to health is outlined as a universal standard by international laws and agreements, current discussions of this concept presume nationality and ignore the vulnerabilities of the 11 to 12 million stateless individuals living worldwide. For those lacking legal status, along with its resulting documentation, medical citizenship is often an impossible goal.

Although framing access to healthcare as a human right is a promising step toward achieving equality, we cannot ignore the importance of nationality as the "right to have rights." In order to move toward universalized access to the right to health, the concept of legal nationality must be considered by scholars, policymakers, and bioethicists. Rather than presuming the existence of nationality, discussants must consider stateless individuals, who have no place to lodge claims for social rights, including the right to health.

### Recommendations

While this paper began as a systematic review of the literature on statelessness and its effect upon access to a supposedly universal right to health, we quickly found that the term, and the concept, is widely absent from the medical literature despite being recognized by human rights specialists as a persistent global problem [4,5,35,36,64]. A search for the term "stateless" on PubMed reveals extremely limited results, while more commonly employed terms such as "refugee" or "internally displaced" reveal a larger body of existent literature. This raises a central point of this discussion: acute humanitarian crises attract the attention of health researchers. However, chronic issues of denationalized, stateless individuals and groups tend to be difficult to find and record, probably due in no small part to weak commitment on the part of host nations to facilitate access to those they have sought to intentionally marginalize and exclude. Our primary recommendation, therefore, is that the global health community begin to recognize the issue of statelessness and its impact upon the ability of those concerned to access healthcare services.

Once statelessness is recognized as an issue that may impact individual and population health, the global health community ought to conduct a great deal more health service research into populations that have been specifically marginalized and excluded from nationality. There is suggestive evidence from NGO field reports, human rights organizations, and limited formal health literature that statelessness is a problem that affects the medical citizenship of individuals around the globe. We hope that those engaged in related research will seek to thoroughly document the problem.

Once identified as a truly significant variable affecting access to healthcare, statelessness must become a priority for the community of global health practitioners, NGOs, and human rights organizations to address directly, alongside acute health crises caused by war or natural disaster. Added advocacy for the healthcare access of stateless persons should also be on the docket. It is one thing for states to recognize a universal right to healthcare by granting such care to their recognized citizens; however, for such a right to be truly universal, nation-states must also grant medical citizenship to those who are presently denied access due to a lack of legal nationality.

## Competing interests

The authors declare that they have no competing interests.

## Authors' contributions

LNK is an interdisciplinary social scientist and a topical expert on statelessness. She was the primary writer of the initial draft of this paper, and participated in all subsequent revisions and discussions. EFC is a political theorist who specializes in citizenship and immigration, and was the primary contributor of the theoretical presentation of citizenship and nationality included in the background sections. She also participated in revisions and discussion of all sections of the manuscript. CPM conceptualized the essay and the need for it in the medical literature. He also contributed content expertise in health policy, and participated in all revisions and discussion of the manuscript. All authors read and approved the final manuscript.

## Pre-publication history

The pre-publication history for this paper can be accessed here:

http://www.biomedcentral.com/1472-698X/10/11/prepub
